# SRSF9 promotes colorectal cancer progression via stabilizing DSN1 mRNA in an m6A-related manner

**DOI:** 10.1186/s12967-022-03399-3

**Published:** 2022-05-04

**Authors:** Xiaoyu Wang, Xiansheng Lu, Ping Wang, Qiaoyu Chen, Le Xiong, Minshan Tang, Chang Hong, Xiaowen Lin, Kaixi Shi, Li Liang, Jie Lin

**Affiliations:** 1 Department of Pathology, Nanfang Hospital, Southern Medical University, Guangzhou, 510515 Guangdong People’s Republic of China; 2 Department of Pathology, School of Basic Medical Sciences, Southern Medical University, No. 1838 Guangzhou Avenue North, Guangzhou, 510515 Guangdong People’s Republic of China; 3 Guangdong Province Key Laboratory of Molecular Tumor Pathology, Guangzhou, 510515 Guangdong People’s Republic of China

**Keywords:** SRSF9, Serine and arginine rich splicing factor 9, DSN1, Component of MIS12 kinetochore complex, *N*6-Methyladenosine (m6A), Epigenetics, Colorectal cancer

## Abstract

**Background:**

Serine/arginine-rich splicing factor 9 (SRSF9) is a classical RNA-binding protein that is essential for regulating gene expression programs through its interaction with target RNA. Whether SRSF9 plays an essential role in colorectal cancer (CRC) progression and can serve as a therapeutic target is largely unknown. Here, we highlight new findings on the role of SRSF9 in CRC progression and elucidate the underlying mechanism.

**Methods:**

CRC cell lines and clinical tissue samples were used. qRT-PCR, Western blotting, immunohistochemistry (IHC), gain- and loss-of-function assays, animal xenograft model studies, bioinformatic analysis, methylated single-stranded RNA affinity assays, gene-specific m6A quantitative qRT-PCR, dual-luciferase reporter assays and RNA stability assays were performed in this study.

**Results:**

The expression level of SRSF9 was higher in CRC cell lines than that in an immortal human intestinal epithelial cell line. Overexpression of SRSF9 was positively associated with lymph node metastasis and Dukes stage. Functionally, SRSF9 promoted cell proliferation, migration and invasion in vitro and xenograft growth. The results of bioinformatic analysis indicated that DSN1 was the downstream target of SRSF9. In CRC cells and clinical tissue samples, the expression of SRSF9 was positively associated with the expression of DSN1. Knockdown of DSN1 partially inhibited the SRSF9-induced phenotype in CRC cells. Mechanistically, we further found that SRSF9 is an m6A-binding protein and that m6A modifications were enriched in DSN1 mRNA in CRC cells. Two m6A modification sites (chr20:36773619–36773620 and chr20:36773645–chr20:36773646) in the SRSF9-binding region (chr20:36773597–36773736) of DSN1 mRNA were identified. SRSF9 binds to DSN1 in an m6A motif- and dose-dependent manner. SRSF9 modulates the expression of DSN1 in CRC cells. Such expression regulation was largely impaired upon methyltransferase METTL3 knockdown. Moreover, knockdown of SRSF9 accelerated DSN1 mRNA turnover, while overexpression of SRSF9 stabilized DSN1 mRNA in CRC cells. Such stabilizing was also weakened upon METTL3 knockdown.

**Conclusion:**

Overexpression of SRSF9 was associated with lymph node metastasis and Dukes stage in CRC. Knockdown of DSN1 eliminated the effects by SRSF9 overexpression in CRC. Our results indicated that SRSF9 functions as an m6A-binding protein (termed “reader”) by enhancing the stability of DSN1 mRNA in m6A-related manner. Our study is the first to report that SRSF9-mediated m6A recognition has a crucial role in CRC progression, and highlights SRSF9 as a potential therapeutic target for CRC management.

**Supplementary Information:**

The online version contains supplementary material available at 10.1186/s12967-022-03399-3.

## Introduction

Colorectal cancer (CRC) is one of the most prevalent malignancies worldwide. It ranks 3rd in terms of incidence and 2nd in terms of mortality among all cancers [1]. In 2020, more than 280,000 people died from CRC in China [2], and the overall 5-year survival rate for patients with advanced CRC is still less than 15% due to metastasis and recurrence [2]. Thus, exploring the molecular mechanism of CRC progression and identifying more specific molecular markers will be helpful in determining early prognosis and progression and will ultimately reduce the mortality caused by this malignancy.

*N*6-Methyladenosine (m6A), the most abundant posttranscriptional modification of mRNA, is emerging as an essential chemical marker that modulates mRNA splicing, maturation, stability and nuclear export and subsequently facilitates translation of mRNA or promotes its turnover [3–8]. m6A mRNA methylation is dynamically catalyzed and regulated by three types of protein complexes: methyltransferases (writers), demethylases (erasers), and binding proteins (readers). “Writers” and “erasers” are responsible for m6A deposition and removal. “Readers” are responsible for recognizing and binding to m6A-contained mRNA and eventually determining the fate of the target mRNAs, thus modulating the physiological and pathophysiological processes that occur in cells. Disrupted “readers” that contribute to incorrect deciphering of the m6A code in mRNA have been implicated in diverse biological processes including tumorigenesis [9]. For example, a group of YTH domain-containing proteins (YTHDFs) have been identified as classical m6A “readers”. High YTHDF1 expression is associated with poor prognosis in hepatocellular carcinoma (HCC) and CRC [10, 11]. YTHDF2 inhibits the proliferation and growth of HCC cells by disrupting the stability of epidermal growth factor receptor mRNA [12], and YTHDF3 negatively regulates the interaction between two long noncoding RNAs, growth arrest-specific 5 (GAS5) and yes-associated protein (YAP) and ultimately inhibits CRC progression [13]. Therefore, the m6A “reader” functions as an oncogene or as an antioncogene in different scenarios that depend on decoding distinct target genes.

Serine/arginine-rich splicing factor 9 (SRSF9), a member of the serine/arginine (SR)-rich family of pre-mRNA splicing factors (SRSFs), constitutes part of the spliceosome and is critical for mRNA splicing [14]. SRSFs also regulate mRNAs by modulating mRNA export from the nucleus and translation [15]. SRSF9 was reported to act as a key regulatory factor by increasing the expression and nuclear accumulation of β-catenin, and a possible mechanism through which it stabilizes β-catenin mRNA and increases its translation was proposed [16]. However, the function of SRSF9 in human cancers, including CRC, has not been studied extensively, and its mechanism of action is even less clear. In 2018, Huang et al. reported that SRSF9 binds to an artificial m6A consensus sequence in high-level [9]. This information gives us a novel perspective from which to investigate the mechanism of action of SRSF9 in human tumors. Here, we focus on the role of SRSF9 in CRC progression and explore whether the underlying mechanism of its effect is related to the functional m6A-binding protein.

## Materials and methods

### Tissue samples and cell lines

Sixty-three paraffin-embedded human CRC tissues and corresponding normal mucosal tissues and sixteen fresh human CRC tissues and matched normal mucosal tissues were collected for this study. All the samples were obtained from the Department of Pathology, Nanfang Hospital, Southern Medical University, Guangzhou, China (from March 2018 to June 2019). All samples were obtained from patients who had been diagnosed with primary CRC by two pathologists based on microscopic analysis of tumor tissue, and none of the patients received radiotherapy or chemotherapy prior to surgical resection. Sixty-three cases, including 38 (60.3%) males and 25 (39.7%) females, were recruited for this study. The use of human materials was approved by the Medical Ethics Committee of Nanfang Hospital, Southern Medical University. Seven human CRC cell lines (HCT8, SW620, Caco2, HT29, HCT15, HCT116, and LOVO) and an immortal human intestinal epithelial cell line (NCM460) were donated by Guangdong Province Key Laboratory of Molecular Tumor Pathology, Southern Medical University and validated by short tandem repeat sequence analysis prior to the start of this project and cultured in Roswell Park Memorial Institute (RPMI) 1640 medium (Gibco, Grand Island, NY, USA) supplemented with 10% fetal bovine serum (FBS, Gibco). All cells were maintained in a cell incubator under 5% CO_2_ at 37 °C.

### Immunohistochemistry (IHC)

IHC was performed according to standard protocols as previously described [17]. After deparaffinization and rehydration, 4-μm-thick tissue slides were blocked with 3% H_2_O_2_ and boiled in ethylenediaminetetraacetic acid (EDTA) (0.01 M, pH 8.0) for antigen retrieval. After blocking with 10% normal goat serum PBS, the slides were incubated with primary antibody at 4 °C, followed by treatment with secondary antibody and diaminobenzidine (DAB). Information on the primary and secondary antibodies used in this study is provided in Additional file 1: Table S1. The staining intensity of each tissue section was determined by two experienced pathologists in a double-blind manner. We referred to published standards to define “low” and “high” expression of SRSF9 [17]. Tumor cells were graded according to the following criteria: ① Staining intensity score: 0, no staining; 1, poor staining; 2, moderate staining; and 3, strong staining; ② Positive staining score: 0, < 10% positive; 1, 10–30% positive; 2, 30–50% positive; and 3, > 50% positive. The total score was calculated as ① Staining intensity score × ② Positive staining score. Samples with total scores ≥ 5 were defined as showing high expression; those with scores < 5 were defined as showing low expression.

### RNA isolation and qRT-PCR

Total RNA was extracted from human CRC cells and tissues, and mRNA was reverse-transcribed into complementary DNA using the PrimeScript™ RT reagent kit (TaKaRa, Shiga, Japan) in accordance with the manufacturer’s recommendations. qRT-PCR was performed in a 7500 Fast Real-Time PCR System with SYBR Green qRT-PCR master mix (TaKaRa). Information on the primer sequences used in this study is provided in Additional file 2: Table S2. Each experiment was performed in triplicate, and expression of all genes was normalized to that of β-actin.

### Western blotting and antibodies

Protein samples were separated by electrophoresis on 10% sodium dodecyl sulfate gels and transferred to polyvinylidene fluoride membranes (Millipore, MA, USA). The membranes were then blocked in 5% skim milk for 1 h at room temperature, followed by incubation with primary antibodies for 8 h at 4 °C. The membranes were incubated with HRP-conjugated secondary antibodies for 1 h at room temperature. Finally, the signal density was detected by chemiluminescence using an ECL reagent kit (FDbio Science, Hangzhou, China). Information on the primary and secondary antibodies used in this study is provided in Additional file 1: Table S1.

### Cell transfection and lentiviral infection

For transient transfection, small interfering RNAs (siRNAs) directed against SRSF9 (#SIGS0008682-4, RIBOBIO, Guangzhou, China), DSN1 (#SIGS0013609-1, RIBOBIO), METTL3 (#SIGS00056532-1, RIBOBIO) and negative control RNAs (si-ctrl) were synthesized by RIBOBIO Company (Guangzhou, China). Transient transfection was performed using a Hieff Trans™ Liposomal Transfection Reagent kit (#40802, Yeasen, Shanghai, China) in accordance with the standard protocol. Cells were collected after 24 h for qRT-PCR and after 48 h for Western blotting and functional studies.

For lentiviral transfection, Flag-SRSF9 (ov-SRSF9), empty vector (Vector), shSRSF9 (sh1, sh2), and shNC were purchased from GeneChem Company (Shanghai, China). Caco2 cells and HT29 cells were used to establish stable SRSF9 overexpression models, and HCT116 cells and LOVO cells were used in the stable SRSF9 knockdown experiments. According to the manufacturer’s instructions, 4 × 10^4^ cells per well were seeded and transfected with the indicated lentiviruses. The infected cells were screened using 5 μg/mL puromycin (Solarbio, Beijing, China) for 1 week or longer, and transfection efficiency was determined by qRT-PCR and Western blotting analysis.

### Cell proliferation assays

For CCK-8 assays, treated cells were placed in 96-well plates at 1 × 10^3^ cells/well in quintuplicate and cultured in RPMI-1640 medium containing 10% FBS for 4 h, 8 h, 16 h, 32 h, and 64 h at 37 °C in 5% CO_2_. CCK-8 detection was performed using a Cell Counting Kit-8 (CCK-8, Yeasen) according to the manufacturer’s instructions. The cells were cultured in the presence of 100 μL of CCK-8 (1:10 dilution) for 2 h at 37 °C, and the optical densities (ODs) at 450 nm of the cultures were then measured. Each sample was analyzed in triplicate.

For the colony-forming assay, treated cells were seeded in 6-well plates at 200 cells/well and incubated in RPMI-1640 medium containing 10% FBS for 2 weeks at 37 °C in 5% CO_2_. The cells were then washed twice with PBS and fixed in 4% paraformaldehyde (Leagene, Beijing, China) for 20 min at 4 °C. Staining with 0.1% crystal violet (Sigma, St. Louis, MO, USA) was then performed within 20 min. The stained cells were counted using a scanner (Bio-Rad, Hercules, CA, USA). Each sample was analyzed in triplicate.

### Transwell migration and invasion assays

Transwell chambers (Corning, NY, USA) were used to observe cell migration and invasion. For migration, cells were collected and resuspended in serum-free medium. A total of 200 µL of cell suspension at 5 × 10^5^ cells/mL was placed in the upper chamber, and 600 µL of RPMI-1640 medium containing 10% FBS was added to the lower chamber. For invasion, the upper chamber was covered with 2 mg/mL Matrigel (Corning) prior to cell seeding. After allowing time for migration and invasion, the cells were fixed in 4% paraformaldehyde (Leagene) for 20 min at 4 °C and stained with 0.1% crystal violet (Sigma) for 30 min. The cells in three randomly selected visual fields of each sample were photographed and counted under a light microscope at 200× magnification. Each sample was analyzed in triplicate.

Wound-healing assay was also performed to observe cell migration. Monolayers of cells were uniformly scratched using the narrow edge of a 10-μL pipette tip and cultured in serum-free RPMI-1640 medium. At 24 h and 48 h thereafter, the extent of healing of the scratches was observed under a light microscope at 200× magnification. Each sample was analyzed in triplicate.

### Flow cytometry

Treated cells were washed three times with PBS and fixed in 70% cold ethanol overnight at 4 °C. The cells were then washed again, centrifuged, suspended and stained with 50 μg/mL propidium iodide and 1 mg/mL RNase in PBS. Cell cycle analysis was performed using a flow cytometer (BD Biosciences, NJ, USA) and analyzed by ModFit software (BD Biosciences). Each sample was analyzed in triplicate.

### In vivo tumorigenesis assay

Animal experiments were approved by the Use Committee for Animal Care and performed in accordance with institutional ethical guidelines for animal experiments. Stable Caco2 and HCT116 cells (5 × 10^6^ cells/injection) were resuspended in 200 μL of PBS and subcutaneously injected into 4-week-old female athymic BALB/c nude mice purchased from the Guangdong Medical Laboratory Animal Center. The resulting tumors were measured every 3–5 days, and their size was calculated according to the formula: volume = 1/2 * (width^2^ × length). After 4 weeks, the mice were sacrificed, and the xenograft tumors were excised, photographed, and fixed in formalin for histological analysis.

### Downstream target prediction

Downstream gene data for the RNA-binding protein SRSF9 were obtained from the web tool POSTAR2 (http://lulab.life.tsinghua.edu.cn/postar), which is a comprehensive database for exploring posttranscriptional regulation based on high-throughput sequencing data and provides information on a large number of binding sites for RNA-binding proteins [18]. Data on the differentially expressed genes (DEGs) between 473 human CRC tissue sample data sets and 41 normal tissue sample data sets downloaded from The Cancer Genome Atlas (TCGA) database (https://www.cancer.gov/about-nci/organization/ccg/research/structural-genomics/tcga) were analyzed using the edge R package [19] of R software (Vienna, Austria). The DEGs between 17 human CRC tissue sample data sets and 17 normal tissue sample data sets obtained from the Gene Expression Omnibus (GEO) database (https://www.ncbi.nlm.nih.gov/geo/) (GSE32323) were analyzed by the limma package [20] of R software. Software default settings were utilized in the analyses. Downstream genes of SRSF9 identified in the POSTAR2 database and the DEGs from TCGA and GEO were screened using Venn diagrams to identify the shared genes. Potential downstream target genes had to meet the following requirements: (1) their mRNAs were binding targets of the human RNA-binding protein SRSF9; and (2) the differences in their expression between human CRC tissue samples and normal tissue samples had P values < 0.05 by statistical analysis and log_2_(fold change) > 1.

### Survival analysis in the CRC dataset

The association of SRSF9 and DSN1 expression with overall survival was analyzed using the web tool GenomicScape (http://www.genomicscape.com/microarray/survival.php), which was established based on data obtained from GEO [21, 22]. Fifty-five CRC individuals in the GEO database (GSE17538) were sorted according to the expression of SRSF9 and DSN1. All cases from GenomicScape were provided to the algorithm for survival analysis. Kaplan–Meier survival plots with hazard ratios (HRs) and log-rank P values were obtained using the webpage. P values < 0.05 were considered to indicate significant differences.

### Methylated single-stranded RNA affinity assay

Single-stranded RNA oligonucleotide probes containing the m6A-binding consensus sequence GGACU with methylated (ss-m6A, 5′-biotin-CGUCUCGG(m6A) CUCGG(m6A)CUGCU-3′) or unmethylated (ss-A, 5′-biotin-CGUCUCGGACUC GGACUGCU-3′) adenosine were synthesized by RIBOBIO Company (Guangzhou, China) and validated by mass spectrometry. According to the standardized procedure used with the RNA pull-down kit (gzscbio, Guangzhou, China), each single-stranded RNA oligonucleotide probe was immobilized on streptavidin magnetic beads and coincubated with total proteins extracted from LOVO cells for 8 h at 4 °C. After two washes, the proteins combined with the single-stranded RNA oligonucleotide probe (methylated or unmethylated) were separated by electrophoresis on 10% sodium dodecyl sulfate gels and detected by silver staining and immunoblotting analysis.

### Silver staining of protein gels

Proteins that had been separated on gels were stained using a Protein Fast Silver Stain Kit (BBproExtra, Guangzhou, China) according to the manufacturer’s recommendations, and the silver signal density was analyzed using a scanner (Bio-Rad).

### Gene-specific m6A qRT-PCR

Total RNA was isolated from LOVO cells. According to the standard operating protocol for the EpiQuik™ CUT&RUN m6A RNA Enrichment Kit (Epigentek, NY, USA), RNA samples were fragmented and incubated with anti-m6A antibody (#A-1801, Epigentek) for 90 min at room temperature. A nonimmune IgG was used as a negative control. RT-qPCR assays with DSN1 primers were performed to quantify the enrichment of m6A-containing RNA. Information on the primer sequences used in this study is summarized in Additional file 2: Table S2. Each experiment was performed in triplicate, and all samples were normalized to β-actin.

### Dual-luciferase reporter assay

cDNAs containing the SRSF9-binding region of DSN1 mRNA sequences (chr20:36773597–36773736) were cloned into a GV361 control reporter plasmid consisting of firefly luciferase (F-luc) and verified by DNA sequencing (GeneChem, Shanghai, China). In the mutant reporter plasmid, adenosine (A) on the m6A motif was replaced by thymine (T). The GV219 vector (GeneChem) formed the backbone of the SRSF9 expression plasmid, and DNA sequencing was performed for verification. 293T cells were seeded into 24-well plates followed by cotransfection with 500 ng of DSN1 luciferase reporter plasmid (GV361-DSN1-WT and GV361-DSN1-MUT) and 0 µg, 0.25 µg, or 0.5 µg of SRSF9 expression plasmid (GV219-SRSF9) using a Dual-Luciferase Assay kit (Promega, WI, USA). After 48 h, the cells were harvested, and luciferase activity was measured according to the recommended protocols. Each group was assayed in triplicate.

### Measurement of mRNA stability

Stable LOVO cells and stable Caco2 cells were incubated with 5 μg/mL actinomycin D (APExBIO, TX, USA) for 0 h, 2 h, 4 h, 6 h, or 8 h, and RNA was then extracted from the cells. Analysis of the half-life of DSN1 mRNA was performed using qRT-PCR as described earlier [9].

### Statistical analysis

The results are presented as the mean ± SD. Student’s t test (two-tailed) and one-way or two-way ANOVA were used to evaluate quantitative data. The chi-squared test was used to analyze qualitative data. The Wilcoxon test was used to analyze rank data. The log-rank test was used to assess significant differences in overall survival. All of the analyses were performed in SPSS 24.0 (SPSS, Inc., IL, USA) and GraphPad Prism 6.0 (GraphPad, Inc., CA, USA). The level of statistical significance was defined as P < 0.05 (*P < 0.05; *P < 0.01; ***P < 0.001; ****P < 0.0001; NS: not significant).

## Results

### SRSF9 is upregulated and associated with poor prognosis in CRC

**Fig. 1:**
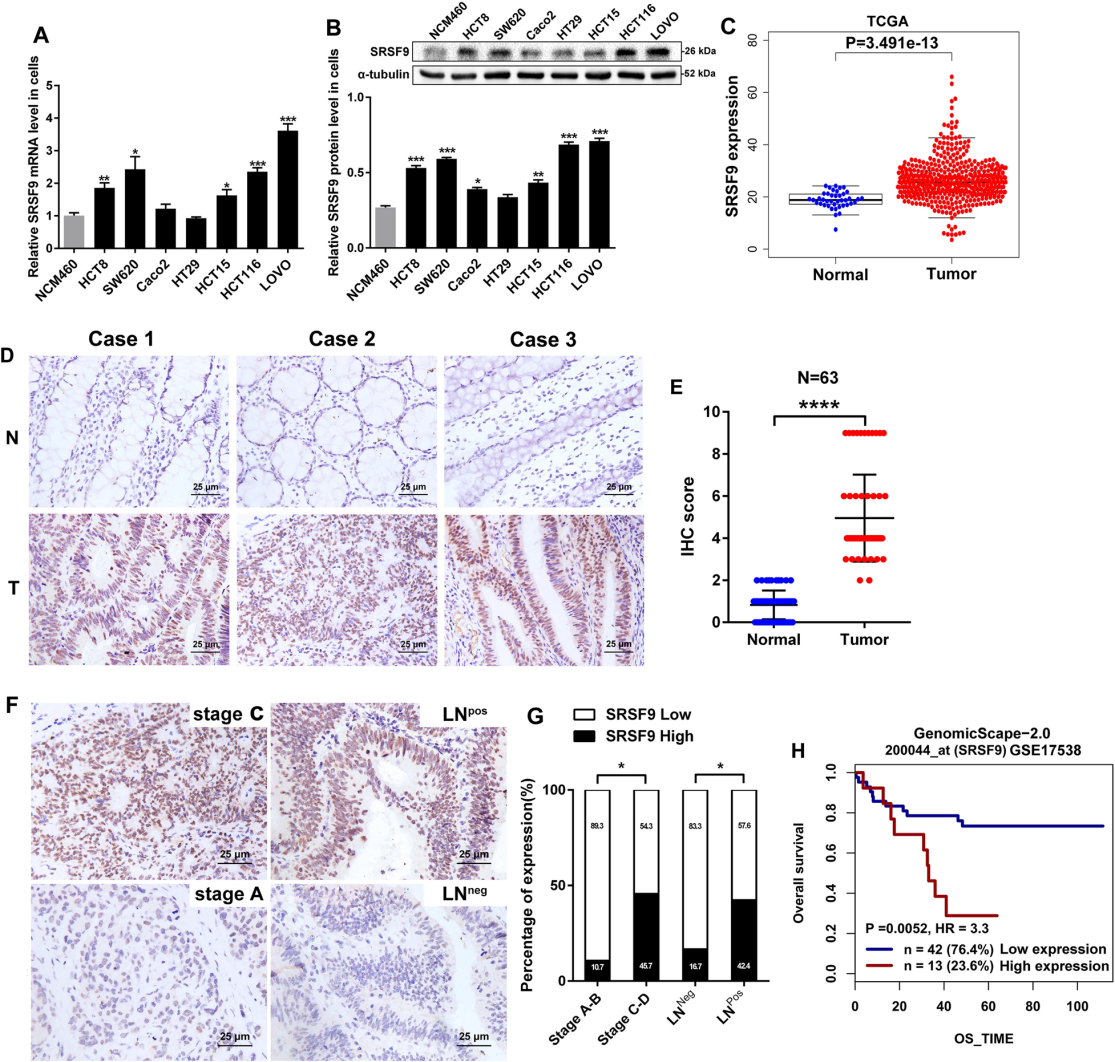
Upregulated SRSF9 expression is associated with poor outcome in individuals with CRC. **A** qRT-PCR assay for detecting relative RNA levels of SRSF9 in CRC cell lines and an immortalized normal intestinal epithelial cell line (*p < 0.05; **p < 0.01; ***P < 0.001, Student’s t test). **B** Western blot assays were employed to detect SRSF9 protein levels in CRC cell lines and in an immortalized normal intestinal epithelial cell line (*p < 0.05; **p < 0.01; ***p < 0.001, Student’s t test). **C** SRSF9 mRNA levels were determined based on the TCGA database (p < 0.05, Wilcoxon test). **D** Representative IHC staining images of SRSF9 staining in CRC tissues and adjacent normal tissues (N: adjacent normal tissues; T: CRC tissues). Scale bar = 25 μm. **E** IHC scores of 63 pairs of CRC tissues based on SRSF9 staining (***p < 0.0001, Wilcoxon test). **F** Representative images showing SRSF9 expression in CRC tumors with different Dukes stages and with or without lymph node metastasis. High expression of SRSF9 was observed for stage C CRC tumors and those that showed positive lymph node metastasis, while low expression of SRSF9 was detected for stage A CRC tumors and those with negative lymph node metastasis (LN^neg^: negative lymph node metastasis, LN^pos^: positive lymph node metastasis). **G** Percentages of cases with high or low expression of SRSF9 according to various clinicopathological features (*p < 0.05, chi-squared test). **H** Survival analysis of CRC patients based on the GEO database (GSE17538) for the correlation between SRSF9 expression and overall survival (p < 0.01, log-rank test). Data are presented as the mean ± standard deviation of the values obtained in three independent experiments

We first measured the expression of SRSF9 in human CRC cells. Our results showed that SRSF9 expression was increased at both the mRNA (Fig. 1A) and protein levels (Fig. 1B) in CRC cells compared to NCM460 cells. We then analyzed the expression of SRSF9 in CRC as reported in TCGA. The results showed that SRSF9 expression was noticeably upregulated in tumor tissues compared with normal tissues (P = 3.491e−13) (Fig. 1C). Furthermore, we analyzed the association between SRSF9 expression and clinicopathological features in 63 CRC patients. IHC assays indicated that SRSF9 protein was mainly localized in the nucleus (Fig. 1D), and the immunoreactivity score revealed that the expression of SRSF9 protein was higher in CRC tissues than in matched normal mucosa tissues (P < 0.001) (Fig. 1E). Overexpression of SRSF9 was significantly associated with tumor lymph node metastasis and high Dukes stage (P < 0.05; Table 1). However, no relationship between SRSF9 expression and other clinicopathological parameters such as sex, age and differentiation grade was found (P > 0.05; Table 1). High SRSF9 expression was more frequently observed in Dukes stage C–D tumors and tumors accompanied by lymph node metastasis than in Dukes stage A–B tumors and tumors not accompanied by lymph node metastasis (Fig. 1F, G). Survival analysis showed that CRC patients (GSE17538) with higher SRSF9 expression exhibited significantly poorer overall survival (OS) (P = 0.0052) and had higher risk of death (HR = 3.3) (Fig. 1H), indicating that CRC patients with elevated expression of SRSF9 generally had poor prognoses. Taken together, our data suggest that SRSF9 expression is elevated in CRC and that it is strongly associated with lymph node metastasis and high Dukes stage. High SRSF9 expression suggests poor prognosis and might be used as an indicator of CRC progression.

**Table 1:** Correlation between SRSF9 expression and the clinicopathological features in 63 CRC patients

Features	SRSF9 expression		χ^2^	P value*
	Low (n, %)	High (n, %)		
Cases	44	19		
Gender				
Male	25 (65.8)	13 (34.2)	0.746	0.418
Female	19 (76)	6 (24)		
Age (year)				
> 45	40 (72.7)	15 (27.3)	1.713	0.229
≤ 45	4 (50)	4 (50)		
Tumor size (cm)				
> 5	14 (73.7)	5 (26.3)	0.191	0.770
≤ 5	30 (68.2)	14 (31.8)		
Grade of differentiation				
Low	6 (85.7)	1 (14.3)	2.270	0.321
Medium	23 (59)	16 (41)		
High	15 (88.2)	2 (11.8)		
Position				
Colon	31 (66)	16 (34)	1.325	0.350
Rectum	13 (81.3)	3 (18.7)		
Perineural invasion				
Yes	9 (69.2)	4 (30.8)	0.003	1.000
No	35 (70)	15 (30)		
Cancer nodules				
Yes	11 (68.8)	5 (31.2)	0.012	1.000
No	33 (70.2)	14 (29.8)		
Tumor depth				
T1–T2	2 (100)	0 (0)	2.527	0.283
T3–T4	42 (68.9)	19 (31.1)		
Lymph node metastasis				
Positive	19 (57.6)	14 (42.4)	4.950	0.031*
Negative	25 (83.3)	5 (16.7)		
Dukes stage				
Stage A–B	25 (89.3)	3 (10.7)	9.204	0.027*
Stage C–D	19 (54.3)	16 (45.7)		

*Chi-square test *p < 0.05

### SRSF9 promotes proliferation, migration and invasion by CRC cells in vitro and in vivo

**Fig. 2:**
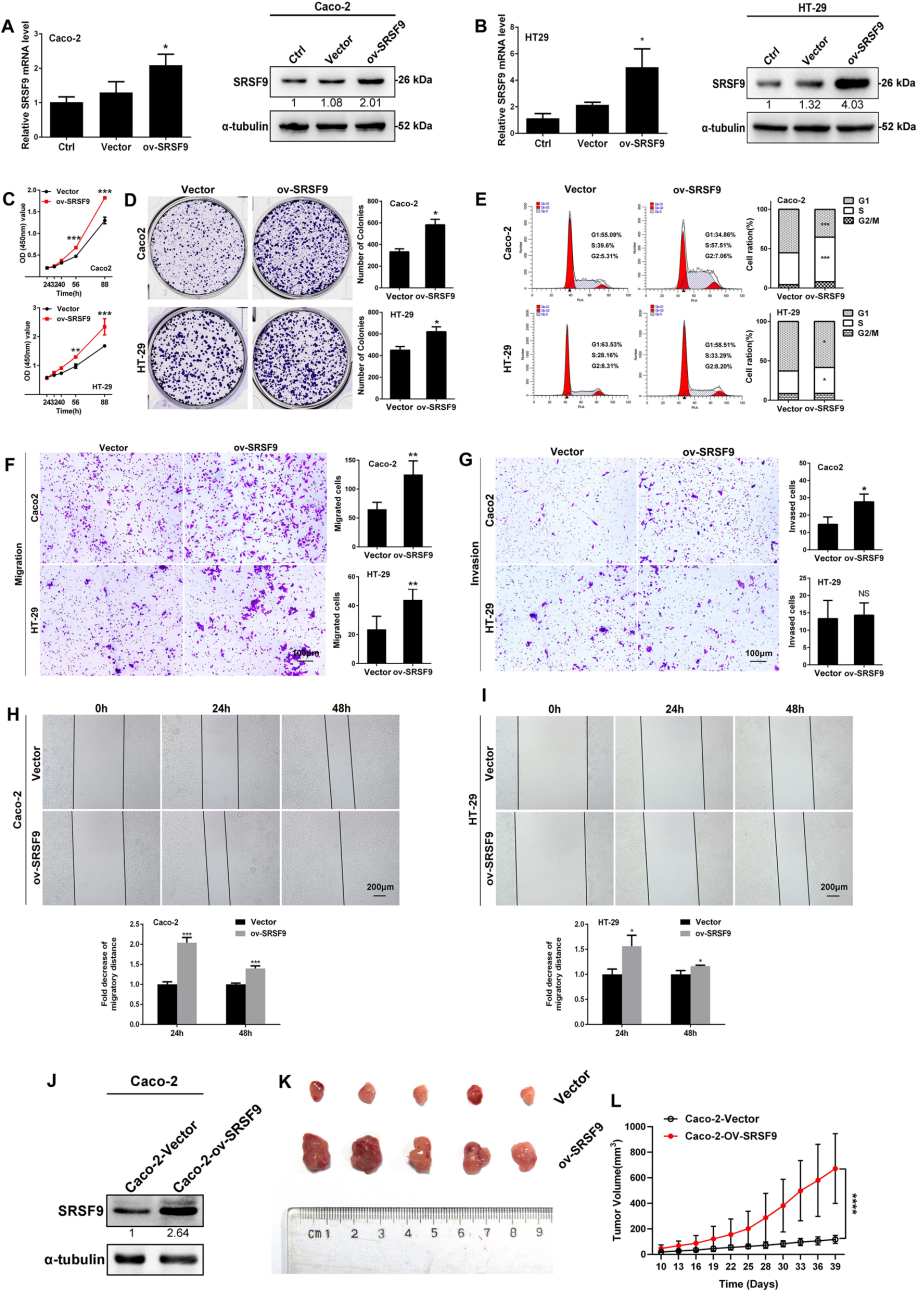
SRSF9 promotes proliferation, migration and invasion by CRC cells in vitro and tumor growth in vivo. **A**, **B** The indicated stable cells were subjected to qRT-PCR (left) and Western blotting (right) (*P < 0.05, Student’s t test). **C**, **D** CCK-8 and colony formation assays were performed in SRSF9-expressing CRC cells (*p < 0.05; **p < 0.01; ***p < 0.001, two-way ANOVA and Student’s t test). **E** Cell cycle distribution was analyzed by flow cytometry in SRSF9-expressing CRC cells (*p < 0.05; ***p < 0.001, one-way ANOVA). **F**, **G** Transwell assays were employed in SRSF9-expressing CRC cells. Scale bar = 100 μm. (*p < 0.05; **p < 0.01; NS: not significant, Student’s t test). **H**, **I** Wound healing assays were performed in SRSF9-expressing CRC cells. Scale bar = 200 μm (*p < 0.05; ***p < 0.001, Student’s t test). **J** The indicated stable cells were subjected to Western blotting. **K** Tumor nodules were collected and used to evaluate the difference in growth that occurred due to the influence of SRSF9. **L** Tumor growth curve based on tumor size measurements (****p < 0.0001, two-way ANOVA). Data are presented as the mean ± standard deviation of the values obtained in three independent experiments

**Fig. 3:**
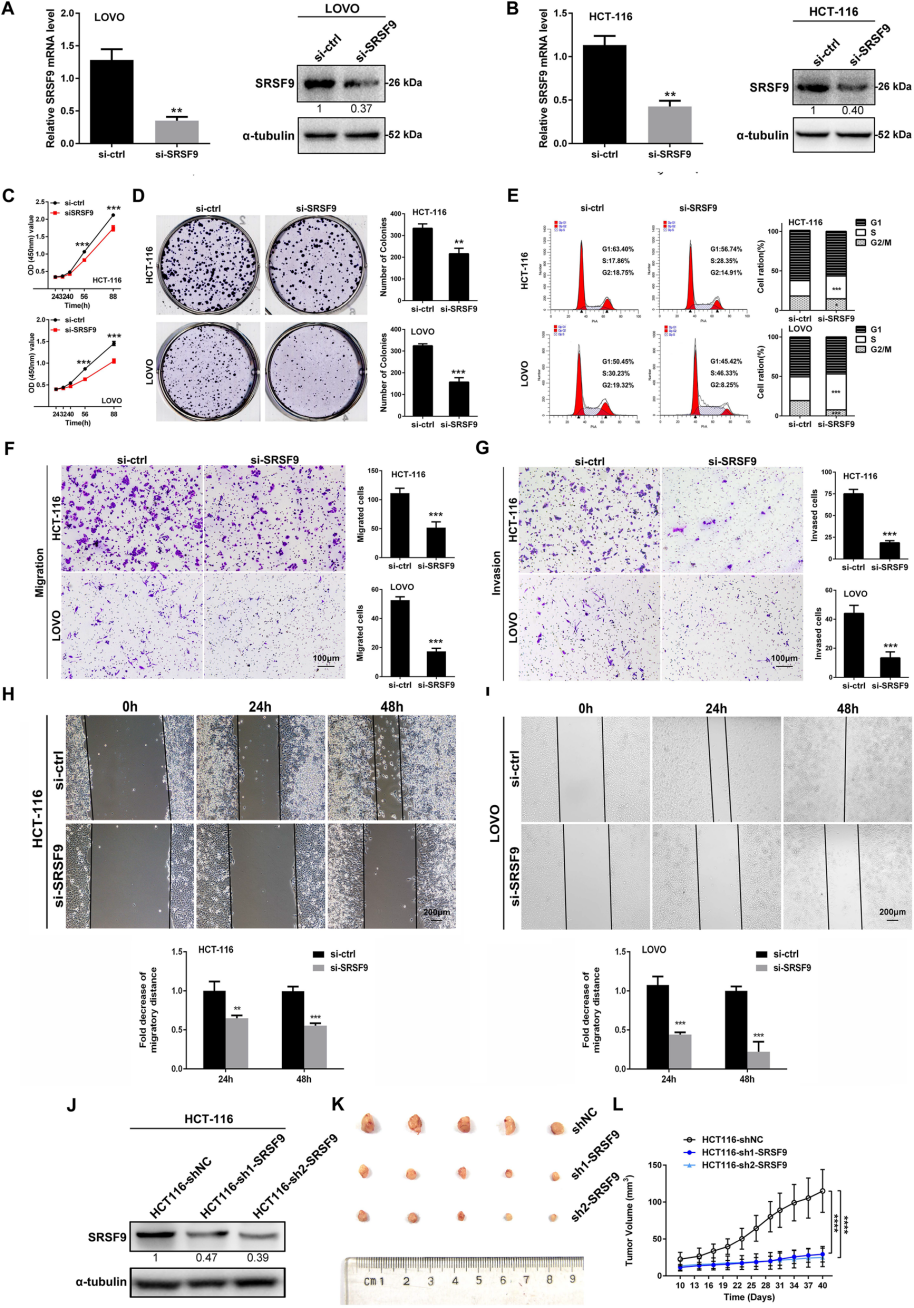
Inhibition of SRSF9 suppresses proliferation, migration and invasion by CRC cells in vitro and tumor growth in vivo. **A**, **B** The indicated infected cells were subjected to qRT-PCR (left) and Western blotting (right). **C**, **D** CCK-8 and colony formation assays were performed in SRSF9 knockdown CRC cells (**p < 0.01; ***p < 0.001, two-way ANOVA and Student’s t test). **E** Cell cycle distribution was analyzed by flow cytometry in SRSF9 knockdown CRC cells (*p < 0.05; ***p < 0.001, one-way ANOVA). **F**, **G** Transwell assays were performed using SRSF9 knockdown CRC cells. Scale bar = 100 μm (***p < 0.001, Student’s t test). **H**, **I** Wound healing assays were performed in SRSF9 knockdown CRC cells. Scale bar = 200 μm (**p < 0.01; ***p < 0.001, Student’s t test). **J** The indicated stable cells were subjected to Western blotting. **K** Tumor nodules were collected and used to evaluate the difference in growth that occurred due to the influence of SRSF9 knockdown. **L** Tumor growth curve based on tumor size measurements (****p < 0.0001, two-way ANOVA). Data are presented as the mean ± standard deviation of the values obtained in three independent experiments

Overexpression and knockdown of SRSF9 were conducted in Caco2/HT29 and LOVO/HCT-116 cells, respectively (Figs. 2A, B and 3A, B). The results of CCK-8 assays (Fig. 2C) and clone formation assays (Fig. 2D) showed that the proliferation ability of CRC cells was increased in the SRSF9-overexpression group compared with the control group, indicating that SRSF9 contributes to the proliferation ability of CRC cells. Flow cytometry showed that compared to that in the control group, the proportion of cells in G0/G1 phase decreased significantly and the proportion of cells in S phase increased in the SRSF9-overexpression group in both cell lines (Fig. 2E), indicating that SRSF9 promotes CRC cell cycle progression. In addition, transwell assays and wound healing assays demonstrated that the migration capability of Caco2 and HT29 cells in the SRSF9-overexpression group was markedly increased (Fig. 2F, H, I), suggesting that SRSF9 enhances the migration ability of CRC cells. Alterations in invasion capability, a characteristic that is related to migration ability, were observed in Caco2 cells but not in HT29 cells (Fig. 2G). This result is probably due to the distinct genetic context that mediates the function of SRSF9 during invasion. Furthermore, as shown in Fig. 2J–L, xenograft tumor burdens were significantly elevated in mice that received subcutaneously transplanted SRSF9-overexpressing Caco2 cells compared to the control group, suggesting that SRSF9 performs a tumor-promoting function in CRC. Taken together, the results of the above gain-of-function assays indicate that overexpression of SRSF9 enhances the growth, migration, and invasion ability of CRC cells in vitro and in vivo.

We also performed loss-of-function assays. The results showed that the growth of LOVO cells and HCT-116 cells in the SRSF9 knockdown groups was noticeably impaired compared with that in the control groups, according to the results of CCK-8 assays (Fig. 3C) and clone formation assays (Fig. 3D). This finding implies that SRSF9 deficiency inhibits CRC cell proliferation. Flow cytometry showed that cell cycle arrest at S phase was induced by SRSF9 knockdown in LOVO and HCT-116 cells (Fig. 3E), suggesting that silencing of SRSF9 can arrest the cell cycle of CRC cells. Cell migration and invasion assays demonstrated that the migration and invasion abilities of LOVO and HCT-116 cells in the SRSF9-inhibited groups were reduced compared to those of the cells in the control groups (Fig. 3F–I), indicating that SRSF9 is necessary for cell migration and invasion in CRC. In addition, tumor xenograft models were constructed by injecting HCT-116 cells with stable knockdown (shSRSF9 sh1, sh2) of SRSF9 into nude mice (Fig. 3J). We found that SRSF9 depletion repressed tumorigenesis (Fig. 3K) and resulted in tumor volumes that were noticeably lower than those in the negative control group (Fig. 3L), suggesting that SRSF9 exerts its oncogenic role in CRC by regulating cell growth in vivo. All the results of the above loss-of-function assays imply that knockdown of SRSF9 represses the proliferation and migration and reduces the invasion capability of CRC cells in vitro and in vivo.

### Identification of SRSF9 targets in CRC

**Fig. 4:**
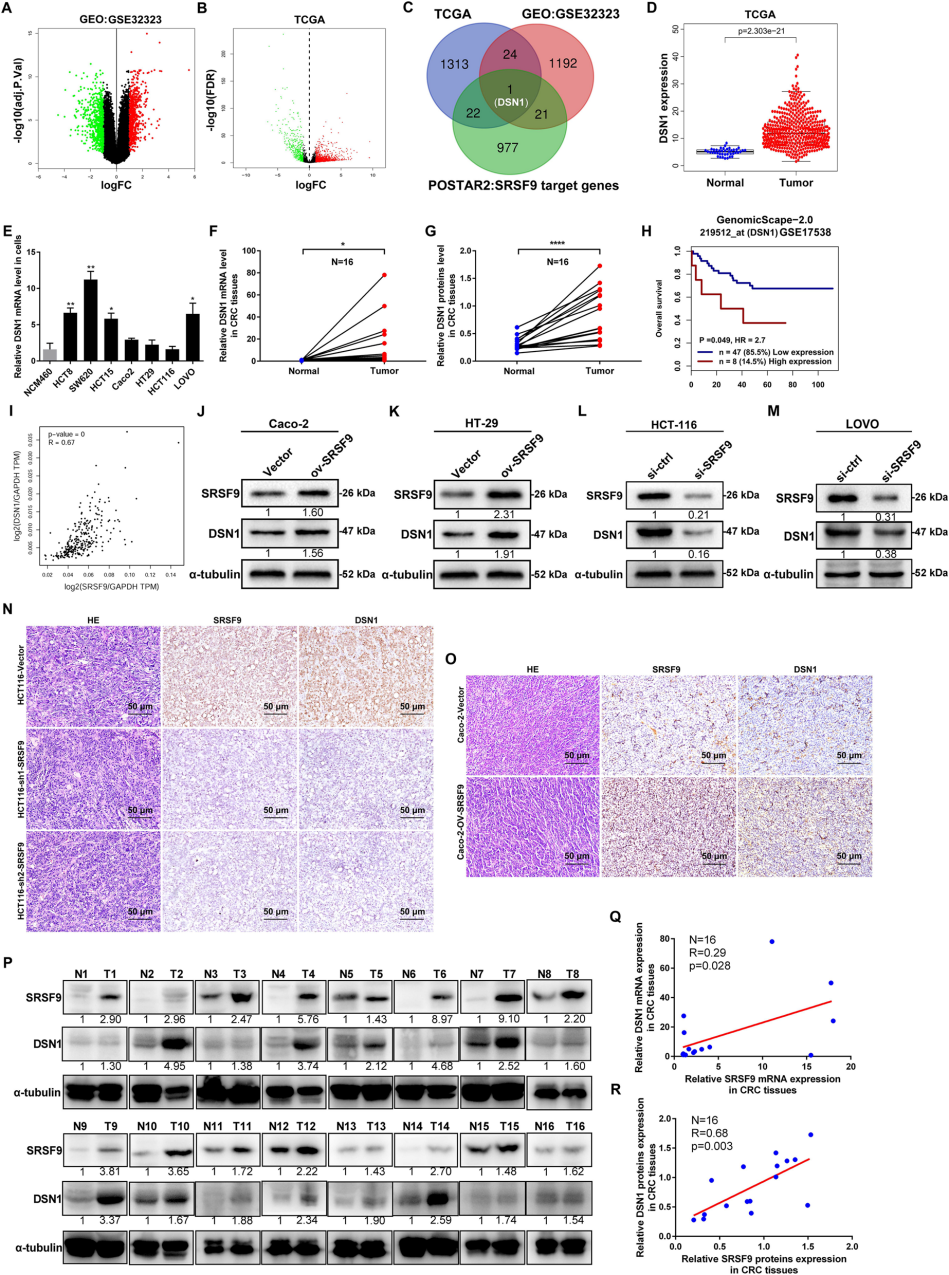
Identification of SRSF9 targets in colorectal cancer. **A** Volcano plots displaying DEGs (1238) between 17 pairs of CRC tissues and adjacent normal tissues from the GEO database (GSE32323) (log_2_(fold change) > 1, p < 0.05). **B** Volcano plots showing DEGs (1360) between 482 CRC tissues and 42 adjacent normal tissues from the TCGA database (log_2_(fold change) > 1, p < 0.05). **C** Venn diagram displaying the overlapping gene (DSN1). **D** DSN1 mRNA levels were determined based on TCGA. **E** qRT-PCR assays were used to measure the RNA levels of DSN1 in CRC cell lines and in an immortalized normal intestinal epithelial cell line. (*p < 0.05; **p < 0.01, Student’s t test). **F**, **G** qRT-PCR (**F**) and Western blot (**G**) assays were used to detect the expression of DSN1 in 16 paired fresh CRC tissues. (*p < 0.05; ****p < 0.0001, Student’s t test). **H** Survival analysis of CRC patients based on the GEO database (GSE17538) for the correlation between DSN1 expression and overall survival (p < 0.05, log-rank test). **I** The expression of SRSF9 and DSN1 was positively correlated in colon adenocarcinoma tumors in the GEPIA database. **J**–**M** Western blot assay showing the protein level of DSN1 after SRSF9 overexpression (**J**, **K**) and knockdown (**L**, **M**) in CRC cells. **(N**, **O)** Representative IHC staining images of SRSF9- and DSN1-positive staining in xenografted tumors. Scale bar = 50 μm. **P**–**R** Western blot assays (**P**, **R**) and qRT-PCR assays (**Q**) were performed to determine the correlation between the expression of DSN1 and that of SRSF9 in 16 pairs of fresh CRC tissues. Data are presented as the mean ± standard deviation of the values obtained in three independent experiments

To understand the mechanism through which SRSF9 exerts its tumor-promoting effects in CRC, we mined POSTAR2 data, GEO data and TCGA data to screen for potential targets of SRSF9 in CRC. The DEGs of CRC tissues and normal tissues obtained from GEO and TCGA are shown in Fig. 4A, B. On systematic screening of the POSTAR2 data, a total of 1021 target mRNAs of the SRSF9 protein were identified (Additional file 3: Table S3). Of these a, 23 were found in both the POSTAR2 data and TCGA data (Additional file 4: Table S4), and 22 were found in both the POSTAR2 data and GEO data (GSE32323) (Additional file 5: Table S5). All these genes are promising targets for SRSF9; we selected DSN1, which is shared by all three profiles, for further study (Fig. 4C).

We first analyzed the expression of DSN1 in CRC according to the TCGA data. The results showed that DSN1 expression was significantly upregulated in CRC tissues compared with normal tissues (P = 2.303e−21) (Fig. 4D). We then performed qRT-PCR and Western blot assays to measure the expression of DSN1 in CRC cell lines and human clinical CRC fresh tissues. The results showed that the level of DSN1 mRNA was upregulated in most CRC cell lines compared with that in NCM460 cell lines (Fig. 4E). Our data for 16 CRC clinical tissue samples also showed that the mRNA (Fig. 4F) and protein (Fig. 4G) levels of DSN1 were noticeably elevated in tumor tissues. Moreover, we performed survival analyses and found that CRC patients with higher DSN1 expression exhibited significantly lower overall survival (P = 0.049) and higher risk of death (HR = 2.7) (Fig. 4H). Thus, the expression of DSN1 is upregulated in CRC patients with a poor prognosis.

The GEPIA database [23] (http://gepia.cancer-pku.cn/) was also used to explore the correlation of the expression of SRSF9 and DSN1 in CRC tissues according to the TCGA data. There was a marked positive correlation between the expression levels of SRSF9 and DSN1 in CRC tissues (R = 0.67, P < 0.001) (Fig. 4I). DSN1 was upregulated in SRSF9-overexpressing CRC cell lines (Fig. 4J, K) but downregulated in SRSF9-inhibited cell lines (Fig. 4L, M) in vitro and in vivo (Fig. 4N, O). In clinical CRC fresh tissue samples, the expression levels of DSN1 and SRSF9 were also positively correlated (Fig. 4P–R). Collectively, our results indicate that there is a positive relationship between the expression of SRSF9 and that of DSN1 in CRC and that alteration of SRSF9 expression induces corresponding changes in DSN1 expression, suggesting that DSN1 is a downstream gene of SRSF9.

### DSN1 deficiency inhibits the tumor promotion effects exerted by SRSF9 overexpression in CRC cells

**Fig. 5:**
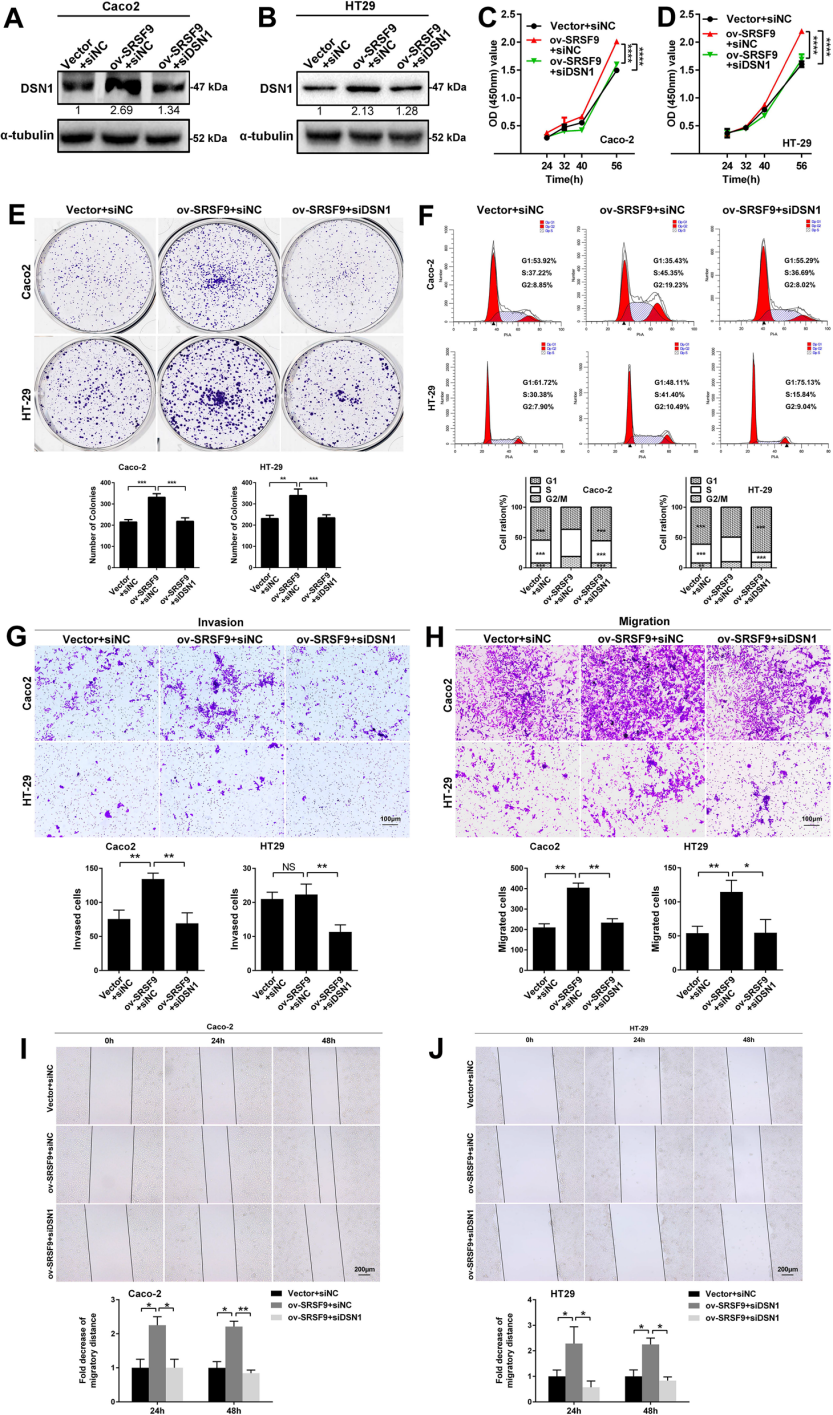
DSN1 deficiency inhibits proliferation, migration and invasion by SRSF9-overexpressing CRC cells. **A**, **B** Western blotting was used to measure the protein levels of DSN1 in DSN1-deficient CRC cells upon overexpression of SRSF9. **C**–**E** CCK-8 assays (**C**, **D**) and colony formation assays (**E**) were performed in the CRC cells described in **A**, **B** (**p < 0.01; ***p < 0.001; ****p < 0.0001, two-way ANOVA and Student’s t test). **F** Cell cycle distribution was analyzed by flow cytometry with CRC cells described in (A-B) (**p < 0.01; ***p < 0.001, one-way ANOVA). **G**, **H** Transwell assays were performed using CRC cells as described in **A**, **B**. Scale bar = 100 μm (*p < 0.05; **p < 0.01; NS: not significant, Student’s t test). **I**, **J** Wound healing assays were performed using CRC cells as described in **A**, **B**. Scale bar = 200 μm (*p < 0.05; **p < 0.01, Student’s t test). Each experiment was performed in triplicate independently

We interfered with DSN1 expression in CRC cells with stable overexpression of SRSF9, which was validated by Western blotting assays before functional experiments were performed (Fig. 5A, B). Notably, SRSF9 overexpression promoted cell growth, colony formation and cell cycle progression in both Caco2 and HT29 cells, whereas knockdown of DSN1 impaired this effect (Fig. 5C–F). DSN1 knockdown also suppressed SRSF9 overexpression-induced cell migration and invasion by CRC cells (Fig. 5G–J). These results suggest that DSN1 is a critical downstream target of SRSF9 that facilitates CRC progression.

### SRSF9 acts as an m6A-binding protein and stabilizes DSN1 mRNA in an m6A-related manner in CRC

**Fig. 6:**
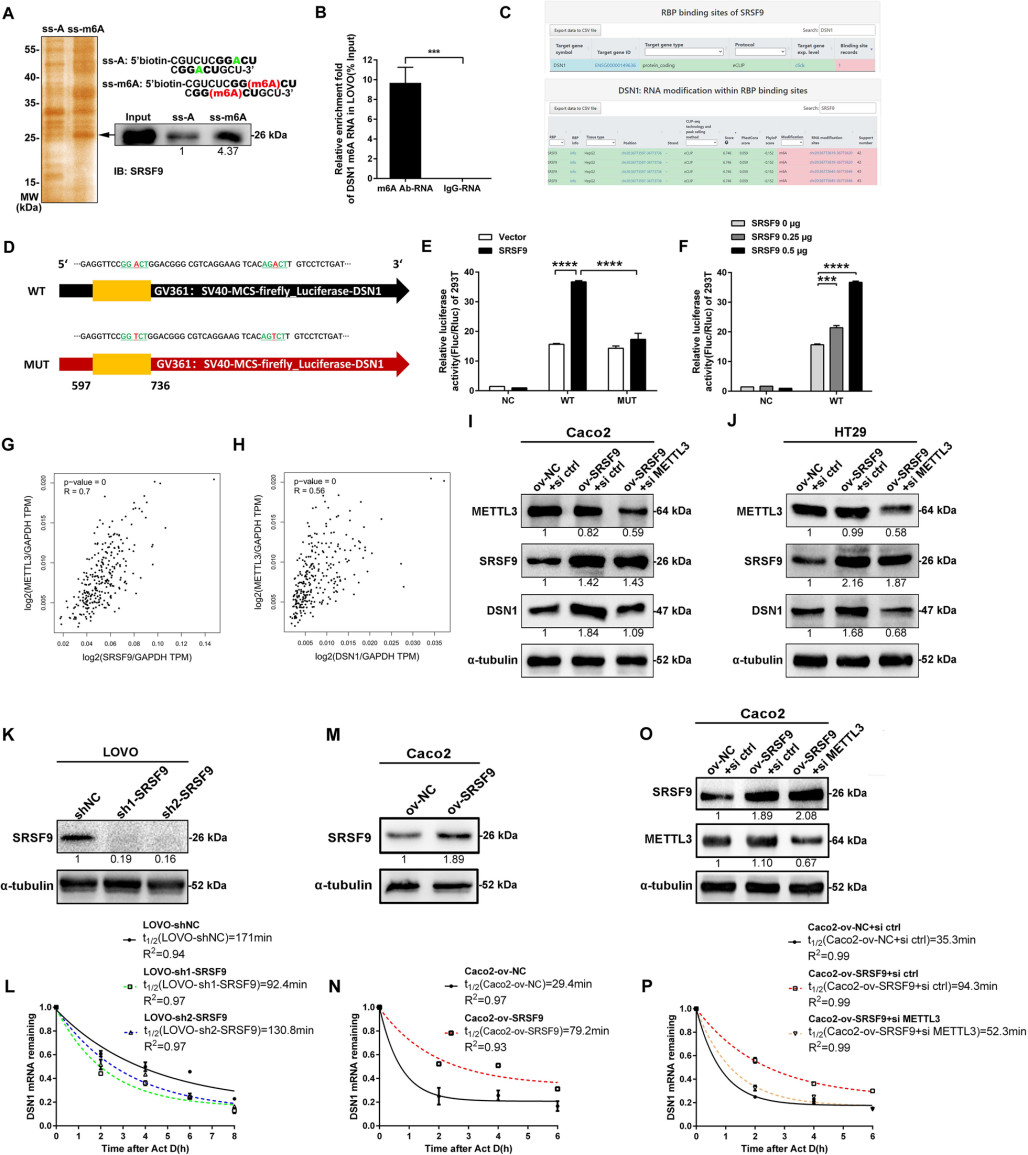
Selective binding of SRSF9 to m6A-modified RNAs stabilizes DSN1 mRNA in an m6A-related manner in colorectal cancer. **A** RNA affinity assay using single-stranded RNA probes containing methylated (red) or unmethylated (green) adenosine. The consensus sequence is shown in bold type. Silver staining (left) and immunoblotting (right) showed selective pull-down of 26 kDa SRSF9 proteins from LOVO cell extracts. IB: Immunoblotting. **B** Gene-specific m6A qRT-PCR was used to detect enrichment of m6A modifications in DSN1 transcripts (***p < 0.001, Student’s t test). **C** Bioinformatic analysis of RNA modifications within the SRSF9 protein-binding sites of DSN1 mRNA. **D** Schematic diagram of the wild-type (WT) and mutant (MUT) firefly luciferase reporters. The A-T substitutions (red) were made within the m6A consensus sequence (green). Only the portion of the SRSF9-DSN1-binding sequence that contains the mutation sites is shown. **E** Relative luciferase activity of WT and MUT reporters in 293T cells ectopically expressing SRSF9 (****p < 0.0001, Student’s t test). **F** Relative luciferase activity of the WT reporter in cells cotransfected with the indicated amounts of the SRSF9 expression vectors (***p < 0.001; ****p < 0.0001, Student’s t test). **G**, **H** The expression of SRSF9 and METTL3 (**G**), DSN1 and METTL3 (**H**) was positively correlated in colon adenocarcinoma tumors in the GEPIA database. **I**, **J** Western blot assay showing the protein level of DSN1 in METTL3-deficient CRC cells upon overexpression of SRSF9 in CRC cells. **K**–**P** The indicated stable cells were subjected to Western blotting (**K**, **M** and **O**). qRT-PCR assays were used to analyze the half-life of DSN1 mRNA in SRSF9 knockdown (**L**), SRSF9-overexpressing (**N**) and SRSF9-overexpressing with METTL3 interfered CRC cells after treatment with actinomycin D (normalized to 0 h). Each experiment was performed in triplicate independently

To identify m6A-binding proteins, we initially used methylated single-stranded RNA bait (ss-m6A) with the consensus sequence GG(m6A)CU or unmethylated control RNA (ss-A) for RNA pull-down to enrich m6A-binding proteins in LOVO cells, followed by immunoblotting and silver staining assays to identify SRSF9 among the m6A-binding proteins. The results showed that the SRSF9 protein binds selectively to the methylated bait (ss-m6A) with an affinity four to fivefold higher than that with which it binds to the unmethylated control (ss-A) (Fig. 6A), suggesting that the SRSF9 protein is an m6A-binding protein. To search for m6A-containing RNA in cells, we performed gene-specific m6A qRT-PCR in LOVO cells using DSN1 mRNA primers. The results showed a significant enrichment of m6A-modified DSN1 mRNA in cellulo (Fig. 6B), indicating that DSN1 mRNA is an m6A-modified transcript.

To explore whether the relationship between SRSF9 and DSN1 is related to m6A modification and recognition, we analyzed the distribution of m6A-enriched regions of DSN1 mRNA across the eCLIP data in POSTAR2. The results showed that DSN1 mRNA is the binding target gene of SRSF9 protein (Fig. 6C upper) and the specific region of DSN1 mRNA to which SRSF9 protein binds contains m6A modifications at two sites (Fig. 6C below). Therefore, we postulated that the association of SRSF9 with DSN1 is correlated with m6A modification. According to the sequence of the SRSF9-DSN1-binding region, dual-luciferase reporter assays with wild-type (WT) and mutant (MUT) plasmids were then conducted. For the MUT reporter, two adenosine bases (A) in the m6A consensus sequences were replaced by thymine bases (T) to eliminate the effect of m6A motifs, while the WT reporter contained the intact m6A sites (Fig. 6D). The results showed that ectopic SRSF9 induced a significant increase in the luciferase activity of the WT reporter (Fig. 6E). Such increases were largely impaired by mutations in the m6A consensus sites (Fig. 6E). Moreover, ectopic SRSF9 induced these increases in a dose-dependent manner (Fig. 6F). Our results demonstrate that the binding of SRSF9 protein and DSN1 mRNA relies on the presence of wide-type m6A motifs within SRSF9-DSN1-binding region. Moreover, TCGA data in GEPIA database showed that there was a marked positive correlation between the expression levels of SRSF9 and methyltransferase METTL3 (R = 0.7, P < 0.001) (Fig. 6G), as well as DSN1 and METTL3 (R = 0.56, P < 0.001) (Fig. 6H) in CRC tissues. The level of DSN1 expression was up-regulated in SRSF9-overexpressing CRC cell lines (Fig. 6I, J). Interference with METTL3 expression in SRSF9-overexpressing cells significantly down-regulated the expression of DSN1 while the level of SRSF9 expression was unchanged (Fig. 6I, J), suggesting that both SRSF9 and METTL3 regulate the expression of DSN1. Given that METTL3 is a typical m6A methyltransferase (termed “writer”) [9], our data indicated that DSN1 is the downstream target of SRSF9 and the regulation of DSN1 by SRSF9 is likely to be related to its RNA m6A modification. Furthermore, we performed mRNA stability assays using SRSF9-knockdown cells (Fig. 6K), SRSF9-overexpressing cells (Fig. 6M) and SRSF9-overexpressing with METTL3 interfered cells (Fig. 6O). Our results showed that the turnover of DSN1 mRNA was markedly accelerated in SRSF9-inhibited cell lines (Fig. 6L), while the half-life of DSN1 mRNA was markedly prolonged in SRSF9-overexpressing cell lines (Fig. 6N) and such stabilizing was weakened upon m6A writer knockdown (Fig. 6P), indicating that SRSF9 promotes the stability of DSN1 mRNA and such regulation is correlated with its RNA m6A modification in CRC cells.

Taken together, our results suggest that SRSF9 acts as an m6A-binding protein by recognizing and binding the m6A modification sites of DSN1 transcripts to enhance the stability of DSN1 mRNA in CRC cells.

## Discussion

CRC has a high prevalence and accounts for approximately 1 in 10 cancer cases and deaths worldwide. In 2020, the incidence and mortality rate of CRC in China ranked third and fifth, respectively, among all malignant tumors [2]. Therefore, it is urgently necessary to improve the early diagnosis and prognosis of CRC by identifying biomarkers and uncovering the molecular mechanisms that contribute to CRC. Epigenetic alterations have emerged as a widespread regulatory mechanism that controls gene expression in diverse pathological processes, especially in cancer pathogenesis and progression. In recent years, RNA modifications have become a hot topic in epigenetic regulation research, among which *N*6-methyladenosine (m6A) modifications are thought to be the most prevalent and abundant modifications that occur in higher eukaryotic mRNAs [24]. Roles for m6A in various diseases have been reported [25, 26]. An increasing number of studies have proposed that m6A-binding proteins (so called “readers”) execute the recognition and interpretation of the target m6A-modified RNA and directly guide various biological processes [27]. For example, m6A modifications in mRNA are recognized by proteins that use the YTH domain (including YTHDF1/2/3 and many more) to bind directly to the m6A motif, thereby promoting the degradation of target RNAs with m6A modifications [28–31]. On the other hand, proteins that belong to the newly discovered category of “reader” proteins, which includes IGF2BP1/2/3, utilize KH domains to recognize m6A-containing transcripts and are responsible for the stabilization of m6A-containing target RNAs [9]. The opposing roles of IGF2BPs and YTHDFs make it necessary for cells to possess alternative mechanisms that permit modulation of downstream target m6A-modified RNAs following m6A modification. In the “m6A-switch” mechanism, the local structure of target m6A-containing RNAs is altered in a way that affects the accessibility of the last type of “readers” to substrates, including HNRNPC, HNRNPG, and HNRNPA2B1. These changes thus regulate the processing of RNA molecules [32, 33]. Together, the characterization of the three main categories of m6A readers deepens our understanding of the effects of m6A-binding proteins on genetic information flow. Thus, it will be interesting to investigate other potential m6A-binding proteins that are able to drive corresponding alterations in genetic messages.

SRSF9, also known as pre-mRNA splicing factor, is a member of the serine/arginine-rich protein family. It is a conserved RNA-binding protein that possesses two RNA recognition motifs (RRMs) and an arginine/serine (RS)-rich domain. The RRMs determine the specificity of the protein’s binding to target mRNAs, and the RS-rich domain is mainly involved in protein–protein interactions [14]. SRSF9 is highly expressed in various malignancies. However, few studies have attempted to determine the biological role of SRSF9 in human cancer. In the present study, our data reveled that SRSF9 was frequently upregulated in CRC cells as well as in clinical CRC tissue samples, and its overexpression was significantly associated with lymph node metastasis, tumor progression and poor prognosis of patients with CRC. These clinical data suggest SRSF9 as a proto-oncogene in CRC, similar to its role in glioblastoma, squamous cell lung carcinoma, malignant melanoma [16] and bladder cancer [34]. Our in vitro and in vivo biological assays also confirmed that SRSF9 promotes CRC proliferation, migration and invasion. These findings further emphasize the core roles of SRSF9 in cancer progression.

Recently, SRSF9 has been shown to bind an artificial m6A consensus sequence with high affinity [9], giving us a distinctive perspective from which to explore its role in human cancers, especially CRC. We attempted to enrich the amount m6A-binding proteins in CRC cells using methylated single-stranded RNA as bait, an efficient and facile test model, with the consensus sequence GG(m6A)CU for RNA pull-down. Among these m6A-binding proteins, SRSF9 was identified by immunoblotting and silver staining. Therefore, we confirmed that SRSF9 is an m6A-binding protein.

The m6A-binding protein (so called “reader”) plays an important role in genetic messages, and dysregulated reader could result in abnormal accumulation of oncogenic products and thereby support the malignant state of cancer. In this study, combining the data on DEGs with the enhanced crosslinking and immunoprecipitation data, we found that DSN1 appeared as the SRSF9 protein high-confidence downstream target in CRC. As the component of MIS12 kinetochore complex, DSN1 plays an important role in sister-mitochondrial interaction including maintaining the stability of the centromere structure, and is also involved in the proper segregation of chromosomes, which is essential for the accurate transmission of genetic material during mitosis [35]. Recently, it has been shown that high expression of DSN1 is associated with chromosomal instability (CIN) [36]. In colorectal cancer, DSN1 affects cell cycle progression and is tightly associated with clinical-pathological features [35]. Furthermore, chromosomal instability, which can lead to abnormal chromosome structure and number, is a prominent feature in human cancers [37]. DSN1 has already been reported to be upregulated and to act as a considerable risk factor in various tumors, and depletion of DSN1 can inhibit the cellular malignant phenotype [35–38]. In our present study, the expression of DSN1 was significantly up-regulated in CRC cells and clinical tissue samples and indicated poor prognosis in CRC patients, suggesting that DSN1 contributes to maintaining the malignant phenotype of CRC. We further found an obvious positive correlation between DSN1 expression and SRSF9 expression both in vitro and in vivo. In addition, overexpression of SRSF9 upregulated the expression of DSN1, while downregulation of SRSF9 contributed to a decrease in the level of DSN1 in CRC cells. The above results indicate that there is an expression correlation between DSN1 and SRSF9 in CRC. Functionally, silencing of DSN1 impaired SRSF9-induced viability, proliferation, cell cycle progression, migration and invasion, indicating that DSN1 and SRSF9 have functional relevance in CRC. Collectively, our results imply that DSN1 is a critical downstream target of SRSF9 that facilitates CRC progression.

Intriguingly, Wu et al. recently reported that MIS12 mRNA, another component of the MIS12 complex that is homologous to DSN1, is highly enriched in m6A modification [39]. In our study, the results of gene-specific m6A qRT-PCR assays confirmed that DSN1 mRNAs were also enriched in m6A modifications in CRC cells. By retrieving eCLIP data in POSTAR2, we found that DSN1 mRNA is the binding target of SRSF9 protein and the information on the binding sequences and distribution of m6A modification sites were also collected. These findings indicate that the interaction between SRSF9 protein and DSN1 mRNA is associated with m6A modification. SRSF9 has been proposed to act through pathways such as the wnt signaling pathway [16] and the apoptosis pathway [34] or through hypoxia-related signaling [40]. However, the involvement of SRSF9 as an m6A-binding protein in modulating downstream targets in human cancer has not been investigated previously. To investigate whether SRSF9 modulates DSN1 in an m6A-related manner, we constructed mutant plasmids using a dual-luciferase reporter system in which the adenosine base (A) in the m6A consensus sites was replaced by a thymine base (T) to eliminate the effect of m6A motifs. Our data reveal that m6A motif within the SRSF9-DSN1-binding region is required for the binding of SRSF9 protein to DSN1 mRNA.

It is well known that methyltransferase-like protein 3 (METTL3) (so called “writer”) is a critical component of the methyltransferase complex, which catalyses methylation at *N*6-adenosine [9]. METTL3 is responsible for the vast majority of m6A sites in mRNA [41]. For this reason, deletion of METTL3 has been used to document numerous m6A-dependent functions [42, 43]. In this study, there is a positive correlation between the expression of METTL3 and SRSF9, METTL3 and DSN1 as well as SRSF9 and DSN1 in CRC. Interference with METTL3 expression in SRSF9-overexpressing cells significantly down-regulated the expression of DSN1 while the level of SRSF9 expression was unchanged. Therefore, we conformed that the expression regulation of DSN1 by SRSF9 is associated with RNA m6A modification. Notably, m6A-binding proteins are pivotal in the stability of their target mRNAs. We speculated that the turnover of DSN1 mRNA in CRC would be affected by SRSF9 in an m6A-related manner. Measurement of the stability of DSN1 mRNA supports our hypothesis. Taken together, these findings indicate that SRSF9 acts in an m6A-related manner in target recognition and regulation in CRC.

## Conclusions

Given the functional diversity of serine-arginine family, the nomenclature of RNA splicing factor likely obscures the broader role of SRSF9 in post-transcriptional regulation of gene expression. Our findings enrich the functional value of serine/arginine family members in hallmarks of tumors. Further investigation is necessary to clarify the molecular mechanism of these events, such as the domain of SRSF9 for m6A recognition and binding, the distribution and enrichment of SRSF9-binding peaks within different gene regions and the profiles of enrichment of SRSF9-binding sites and m6A modifications across mRNA transcriptome. This study also illustrates that the kinetochore-associated protein DSN1 is a critical downstream target of SRSF9. Additional studies involving other types of cancer may be undertaken to explore whether the m6A-related manner through which SRSF9 targets DSN1 could be broadly applied as a potential basis for cancer therapy.

## Supplementary Information

**Additional file 1: Table S1.** Summary of information of antibodies.

**Additional file 2: Table S2.** The primer sequences used for RT-qPCR.

**Additional file 3: Table S3.** Target mRNAs of the SRSF9 protein in POSTAR2.

**Additional file 4: Table S4.** The differentially expressed genes (DEGs) between 473 human CRC tissue sample data sets and 41 normal tissue sample data sets in The Cancer Genome Atlas (TCGA) database.

**Additional file 5: Table S5.** The DEGs between 17 human CRC tissue sample data sets and 17 normal tissue sample data sets in the Gene Expression Omnibus (GEO) database.

## Data Availability

All data generated or analysed during this study are included in this published article.

## Abbreviations

m6A: *N*6-Methyladenosine
CRC: Colorectal cancer
TCGA: The cancer genome atlas
GEO: Gene Expression Omnibus
GEPIA: Gene Expression Profiling Interactive Analysis
DEGs: Differentially Expressed Genes
OS: Overall Survival
IHC: Immunohistochemistry
CCK-8: Cell Counting Kit-8
qRT-PCR: Quantitative reverse transcription polymerase chain reaction
