# Supplementary material for: SRSF9 promotes colorectal cancer progression via stabilizing DSN1 mRNA in an m6A-related manner
Source: J Transl Med. 2022 May 4;20:198. doi: 10.1186/s12967-022-03399-3 (PMC9066907; doi:10.1186/s12967-022-03399-3)
Supplement: Supplementary file 1 — Additional file 1: Table S1. Summary of information of antibodies. [file 12967_2022_3399_MOESM1_ESM.doc]

**Table S1. Summary of information of antibodies**

| **Name** | **Supplier** | **Catalog number** | **Host**  **species** | **Application** | **Dilution** |
| --- | --- | --- | --- | --- | --- |
| Primary antibodies |  |  |  |  |  |
| SRSF9 | Abcam | #ab236414 | mouse | IHC-P  WB | 1:150  1:2000 |
| DSN1 | Invitrogen | #PA5-51742 | rabbit | IHC-P  WB | 1:200  1:250 |
| METTL3 | Abcam | #ab240595 | rabbit | WB | 1:2000 |
| α-tubulin | Proteintech | #11224-1-AP | rabbit | WB | 1:1000 |
| Secondary antibodies |  |  |  |  |  |
| EliVisonTM Plus mouse/rabbit kit | MAXIM biotechnologies | KIT-9902 | - | IHC-P | Ready-to-use |
| anti-mouse IgG -HRP | ABclonal | #AS003 | goat | WB | 1:2000 |
| anti-rabbit IgG -HRP | ABclonal | #AS014 | goat | WB | 1:2000 |

IHC-P, immunohistochemistry-paraffin; WB, western blot
