# Supplementary material for: SRSF9 promotes colorectal cancer progression via stabilizing DSN1 mRNA in an m6A-related manner
Source: J Transl Med. 2022 May 4;20:198. doi: 10.1186/s12967-022-03399-3 (PMC9066907; doi:10.1186/s12967-022-03399-3)
Supplement: Supplementary file 2 — Additional file 2: Table S2. The primer sequences used for RT-qPCR. [file 12967_2022_3399_MOESM2_ESM.doc]

**Table S2. The primer sequences used for RT-qPCR**

| **Genes** | **Sequence (5’ to 3’)** |
| --- | --- |
| hSRSF9-F | CATGGAATATGCCCTGCGTAAAC |
| hSRSF9-R | ACCGTGAGTAGCCATAGCTGGTG |
| hDSN1-F | GCTGCAGGCCTTTATGGATGA |
| hDSN1-R | CCATGTATGGCAGGTGGGTTC |
| hβ-actin-F | CTCCCTGGAGAAGAGCTACGAGC |
| hβ-actin-R | CCAGGAAGGAAGG CTGGAAGAG |
